# Hereditary diseases and child wish: exploring motives, considerations, and the (joint) decision-making process of genetically at-risk couples

**DOI:** 10.1007/s12687-021-00510-x

**Published:** 2021-02-20

**Authors:** Y. Severijns, C. E. M. de Die-Smulders, T. Gültzow, H. de Vries, L. A. D. M. van Osch

**Affiliations:** 1 Department of Health Promotion/CAPHRI, Maastricht University, PO Box 616, 6200 MD Maastricht, The Netherlands; 2 GROW School for Oncology and Developmental Biology, Maastricht University Medical Centre+, Maastricht, The Netherlands; 3 Department of Clinical Genetics, Maastricht University Medical Centre +, Maastricht, The Netherlands

**Keywords:** Hereditary diseases, Child wish, Joint decision-making, Preimplantation genetic testing (PGT), Prenatal diagnosis, Genetic counseling

## Abstract

Couples who are at risk of transmitting a genetic disease to their offspring may face difficult challenges regarding reproductive decision-making. Deciding if, and how, to purse their child wish can be a demanding process. This study aims to describe the reproductive joint decision-making process of genetically at-risk couples. A qualitative study was conducted with 16 couples (*N*=31) at risk of transmitting a genetic disease to their offspring and who received genetic counseling. Most couples were not aware of all available reproductive options in the Netherlands. A variety of motives was reported with almost all couples expressing a preference towards a reproductive option in which the child is genetically related to both parents. Only a few couples considered other options such as the use of donor gametes, adoption, and foster parenting. All couples indicated that they had multiple conversations to reach a mutually supported reproductive decision. Several carriers reported feelings of guilt and in some couples, the woman appeared to have a greater impact in the decision-making process as she should carry a pregnancy and should undergo medical treatments. This study provides insight in the extensive decision-making process of genetically at-risk couples and the role of both partners in this process. These findings can guide the development of genetic counseling (e.g., increase awareness of available reproductive options) and decision support for these couples.

## Background

Couples at risk of transmitting a genetic disorder to their offspring may encounter challenges when deciding if and how to fulfil their child wish (Reumkens et al. 2018). They have several reproductive options. In three of them, the child is genetically related to both parents: natural conception without genetic testing, prenatal diagnosis (PND), and preimplantation genetic testing (PGT) (Cunningham et al. 2015). Opting for natural conception without genetic testing implies acceptance of the risk of transmitting the disease to their offspring (Cunningham et al. 2015). This risk of an affected child depends on the mode of inheritance, and is 50% for autosomal dominant disorders (e.g., Marfan syndrome and Huntington’s disease), 25% for autosomal recessive disorders (e.g., sickle cell disease and cystic fibrosis), and 25% for female carriers of X-linked disorders (e.g., hemophilia) (De Krom et al. 2015; Genetic and District of Columbia Department of 2010; Genoff Garzon et al. 2018; Henneman et al. 2001). Couples with chromosomal translocations face a variable risk of a miscarriage and an ongoing pregnancy of offspring with an unbalanced chromosomal anomaly (De Krom et al. 2015). PND involves genetic testing of the fetus during pregnancy. If the fetus is affected, couples can opt for termination of pregnancy (TOP) or prepare for having a child with the disease (De Die-Smulders et al. 2013). PGT involves genetic testing of in vitro fertilized (IVF) embryos. Only embryos predicted to be unaffected are transferred into the uterus (Järvholm et al. 2018; Rich et al. 2014). Couples may use PGT to circumvent the difficult decision of terminating a pregnancy as is the case in PND (Genoff Garzon et al. 2018). Couples can also opt to have non-genetically related children (adoption or foster parenting) or use donor gametes. In the latter case, only one of the parents will be genetically related to the child. However, uptake of these latter reproductive options is generally low, most likely because couples prefer to have a genetically related child and because of the complexity of the procedures (De Die-Smulders et al. 2013; Evers-Kiebooms et al. 2002; Quinn et al. 2010; Richards and Rea 2005; Schover 2009). Lastly, couples may also decide to refrain from having children (Myring et al. 2011).

Previous studies have shown that couples experience difficulties in the reproductive decision-making process (Gietel-Habets et al. 2018; Klitzman et al. 2007). Couples weigh their needs, desires, and competing concerns (e.g., physical and psychological concerns, e.g., regarding an IVF treatment) and they report difficulties resolving these concerns (Gietel-Habets et al. 2018; Klitzman et al. 2007). Additionally, couples may experience feelings of doubt and uncertainty regarding their decision, which sometimes remained for years after making a reproductive decision (Derks-Smeets et al. 2014; Quinn et al. 2010). Apart from the risk of transmission of the disease, disease characteristics such as age of onset, lethality, and (perceived) severity contribute to the complexity of this process (van Rij et al. 2011). Furthermore, reproductive decision-making affects not only the lives of couples but also the lives of their future offspring. This may raise concerns regarding the profound meaning and long term consequences of their decision, such as the health of their future child(ren) (Hershberger et al. 2012). The process of reproductive decision-making can therefore be demanding and raise emotional, cognitive, and practical questions. Careful deliberation of perceived advantages and disadvantages of different reproductive options and the concordance of options with personal values is therefore advocated (Derks-Smeets et al. 2014).

Deciding on a reproductive option is a major life choice for couples. However, little is known about the motives and considerations of couples to choose for or refrain from these options, nor about the decision-making process *between* partners. Most previous studies that explored reproductive decision-making mainly focused on specific hereditary diseases (Gietel-Habets et al. 2017; Kazmerski et al. 2017; Klitzman et al. 2007). When making health care decisions, the decisional partner can contribute as a source of support or conflict in the decision-making process (Gray et al. 2019). In this process, couples can have conflicting views and different opinions regarding reproductive options (Anderson 2007; Klitzman et al. 2007; Myring et al. 2011). Although previous research indicates that active participation by both partners in a decision-making process may lead to better outcomes, little is known about the role of both partners in the reproductive decision-making process and how they communicate about their views regarding different reproductive options (Osamor and Grady 2018). Previous research regarding prenatal screening has shown that the input of the partner was considered as most important compared to the input of others and that couples think they should make the decision jointly (Carroll et al. 2012; Laberge et al. 2019). More research is necessary to explore the joint decision-making process regarding reproductive options to find leads on how to support couples in this process.

In order to support couples in their reproductive decision-making, recently, an online decision aid was developed for couples with hereditary cancer (Reumkens et al. 2019a). Couples need to have accurate and balanced information to make an informed decision (Raffle 2001). As reproductive motives are likely to be different between various types of genetic conditions, we will extend this online decision aid to fit the needs and wishes of all couples at risk of transmitting other genetic diseases to their offspring. Therefore, the primary aim of this study is to provide insight into different motives and considerations of couples with different genetic diseases and inheritance patterns. The second aim of this study was to explore the joint decision-making process of these couples to gain better insight in the way couples communicate about and decide on reproductive options.

## Methods

A qualitative study was conducted using semi-structured interviews among couples at increased risk of transmitting a genetic disease to their offspring. The study was evaluated and approved by the medical ethical committee of the Maastricht University Medical Centre (MUMC+ 11-4-065).

### Participants and procedures

Participants were recruited from March 2019 to October 2019 by health professionals of the clinical genetics department of MUMC+ and by online messages on websites of two patient federations (Dutch Patient Alliance for Rare and Genetic Diseases and Contact group Marfan Netherlands). Couples were eligible for participation if they had an increased risk of transmitting a genetic disease to their offspring, e.g., if one partner is or if both partners are a proven carrier(s) of a genetic disorder or chromosomal abnormality, if both partners were 18 years or older, if they had received genetic counseling regarding their child wish within the previous 2 years, if they had made a reproductive decision, and if they had sufficient knowledge of the Dutch language. Eligible couples were informed about the study by their treating physician. If they express interest in this study, they received a patient information letter for participation (including the goal of the research) and an informed consent form for each partner by mail. If they definitely decided to participate, they signed the informed consent form. Couples who were recruited by online messages contacted the researcher by e-mail and showed their interest to participate in this study. Afterwards, they received a patient information letter for participation and an informant consent form for each partner by mail. Couples were contacted by phone or e-mail by the researcher to schedule an appointment to conduct the interview. Participants were interviewed at a location of their preference, mostly in their home environment, and received a gift card of 20€ for their participation.

In order to reach heterogeneity of participants with regard to genetic disease and inheritance patterns, and reproductive decisions, purposive sampling was conducted. We included couples who had opted for natural conception without genetic testing, PGT, PND, adoption, foster parenting, the use of donor gametes and refraining from having (further) children.

### Interviews

Before the start of the interviews, participants were asked to complete a brief questionnaire on demographic characteristics (e.g., age, education) and their genetic status (e.g., genetic disease, affected, or carrier). An interview route was used to provide guidance throughout the semi-structured interviews. Several experts in qualitative research, psychology, and clinical genetics provided feedback on the interview route, and improvements were made accordingly, resulting in a final version. The interview guide focused on motives and considerations of couples with regard to the various reproductive options and the process of decision-making. Participants were asked to introduce themselves and elaborate on their reproductive history. Thereafter, participants were asked about factors that had influenced their decision-making and the process of (joint) decision-making. They were asked about their perceived advantages and disadvantages of reproductive options and which reproductive options they had considered. They were also asked whether they had made their decision primarily based on cognition (i.e., rational thoughts, knowledge or objective beliefs, or affect (i.e., emotions, feelings, or gut-feelings)). Finally, couples were invited to elaborate on the process of decision-making, i.e., how often they discussed the topic with their partner, whether they found it difficult to make a decision, to what extent the decision was made jointly, and what role each of the partners fulfilled in the decision-making process. One moderator (Y.S., female PhD student, trained in qualitative research) conducted all interviews.

### Data analysis

All interviews were audiotaped, anonymized, transcribed verbatim, and analyzed by using NVivo 12. Two researchers (Y.S. and T.G.) analyzed 30% of the interviews by deductive and inductive coding to ensure reliability. Deductive coding refers to codes that were generated a priori based on previous research and theoretical insights. First, Y.S. developed a coding tree that was based on themes known from previous research, e.g., categories of motives and considerations (Derks-Smeets et al. 2014). The coding tree consisted of major and minor themes related to the interview questions and was perceived to cover all the relevant information. The coding tree was subsequently applied to one randomly selected transcript by both researchers. Additionally, inductive codes were assigned to new themes (e.g., feelings of guilt) that emerged from the data. During this process, inconsistencies in coding were discussed and agreed upon by both Y.S. and T.G. This resulted in a final coding tree. Subsequently, both researchers coded four transcripts and intercoder reliability was assessed. Cohen’s Kappa of 0.80 was reached, which can be regarded as a strong level of intercoder agreement (McHugh 2012). Subsequently, the remaining 12 transcripts were coded by one researcher (Y.S.). The brief questionnaires were analyzed by descriptive statistics using SPSS (version 24).

## Results

### Couples’ characteristics

Of the 19 couples that received the information, 15 opposite-sex couples and one single woman (*N*=31) agreed to take part in the interview (participation rate 84%). In the remainder of this manuscript, we will use the term “couples,” also when referring to the single woman. Reasons for non-participation were experiencing a difficult time because of private reasons, lack of time, and feeling uncomfortable with an interview. The interviews were conducted between May and October 2019 and lasted between 40 and 80 min. Couples with different inheritance patterns were included, couples were at risk of transmitting an autosomal dominant disorder (*N*=8), an autosomal recessive disorder (*N*=4), X-linked disorder (*N*=1), high risk of genetic disorder, unknown gene defect (*N*=2), and chromosomal abnormality (*N*=1). Almost all couples already implemented their decision, except for one couple who was waiting for PGT approval. Couples opted for natural conception (*N*=3), PND (*N*=3), PGT (*N*=3), donor gametes (*N*=3), adoption/foster parenting (*N*=2), and remaining childless (*N*=2). The majority of the participants have made their decision in the last 2 years. Some couples made their decision up to 5 years ago. Participants’ characteristics are summarized in Table 1 and the reproductive history in Table 2.

**Table 1:** Couples’ demographic characteristics

Characteristic	*N*	Percentage
Gender		
Male	15	48.4
Female	16	51.6
Mean age		
Male	34.9 (SD = 5.7)	
Female	33.9 (SD = 6.1)	
Education^1^		
Low	5	16.1
Middle	9	29.0
High	17	54.8
Inheritance pattern		
Autosomal dominant	8	50.0
Autosomal recessive	4	25.0
X-linked	1	6.25
Chromosomal	1	6.25
Unknown gene defect	2	12.5
Carrier		
Male	3	18.75
Female	8	50.0
Both	5	31.25
Reproductive decision		
Natural conception	3	18.75
PND	3	18.75
PGT	3	18.75
Donor gametes	3	18.75
Adoption/foster parenting	2	12.25
Remaining childless	2	12.25
Made their reproductive decision		
< 1 year ago	6	37.5
< 1–2 years ago	3	18.75
< 2–5 years ago	4	25%
> 5 years ago	3	18.75

^1^*Low*, less than primary, primary, and lower secondary education; *middle*, upper or post-secondary non-tertiary education; *high*, tertiary education (Reumkens et al. 2019b)

**Table 2:** Couples’ reproductive history

Couples’ code	Inheritance pattern^1^	Unaffected child^2^	Live born affected child	Neonatal death	Miscarriages	TOP of the affected child	Pregnant at the time of interview	Reproductive choice^3^
1	U	1	-	1	-	-	-	DG
2	U	1	-	1	-	1	-	DG
3	AD	-	-	-	-	-	-	R
4	AD	-	-	-	-	-	-	PGT
5	AD	-	-	-	-	-	-	PGT
6	AR	1	-	1	-	1	-	PND
7	C	1	-	-	1	2	-	A/F
8	AD	-	2	-	-	-	-	NC
9	AR	1	-	-	-	-	-	DG
10	AD	-	-	-	-	-	1	PND
11	X	1	-	-	-	-	-	A/F
12	AR	-	-	-	-	-	-	PGT
13	AD	-	-	-	-	-	-	R
14	AD	-	-	-	1	-	-	PND
15	AR	-	1	-	1	-	-	NC
16	AD	1	-	-	-	-	-	NC

^1^*U*, unknown gene defect; *AD*, autosomal dominant;*AR*, autosomal recessive; *C*, chromosomal; *X*, X-linked

^2^This includes unaffected children after natural conception, PND, PGT, and the use of donor gametes

^3^This is the reproductive choice couples were interviewed about, the most recent choice they made. *DG*, donor gametes; *R*, refraining from having (a) child(ren); *PGT*, preimplantation genetic testing; *PND*, prenatal diagnosis; *A/F*, adoption or foster parenting; *NC*, natural conception without genetic testing

### Consideration of options

Couples were asked to name all the reproductive options they know. All couples were aware of the reproductive options PGT and natural conception without extra genetic testing. Almost all (*N*=14) couples mentioned adoption, some couples mentioned PND (*N*=10) and the use of donor gametes (*N*=6), and only a few mentioned foster parenting (*N*=2) and remaining childless (*N*=3) and the use of donor gametes (*N*=6). Fourteen couples indicated that they had not considered remaining childless as an option. They would only consider this if the other options would be unsuccessful.

## Motives and considerations regarding different reproductive options

### PGT

The main motive mentioned by all couples who opted or considered opting for PGT was the desire to have a healthy child. They want to protect their child from the impact of the disease based on their personal and/or family experiences with the disease. Male partner 4: “You want the best for your children and if there is a possibility for this, why shouldn’t you try this?” Additionally, many couples mentioned their wish to have a child that is genetically related to both of them. Female partner 12: “Before I was aware of PGT, I always assumed that we could never have a child together.”

Several couples wanted to avoid feelings of guilt towards their future children. Female partner 5: “I do not blame my parents for this, they were not aware of the disease. However, I do not want that my child would blame me for having the disease.” Another motive mentioned by couples was the concern that their children should face the same reproductive dilemma as they did and they wanted to avoid this. Relatedly, couples mentioned that they wanted to wipe out the mutation in their family line. Female partner 4: “I do not want that our children should face the same reproductive dilemma; the disease ends here.”

Compared to PND, preventing a possible termination of pregnancy was also perceived as an advantage by several couples. Female partner 15: “PGT does not feel as a termination of a pregnancy, it feels like choosing which pregnancy it will be.” However, couples mentioned ethical issues regarding PGT related to the moral duty to protect the child and the nature of the condition, which was influenced by the severity of the disease. Female partner 4: “Can I make the choice that a child cannot live with this disease? Am I the person who can decide on this?”

Couples also mentioned several disadvantages of PGT, mainly practical issues. Reasons included the long duration of the trajectory and the frequent hospital visits. Female partner 12: “Before you become pregnant, it will take at least one or even two years. That creates a lot of time pressure.” Additionally, the perceived low chance of a successful pregnancy was mentioned as a disadvantage of PGT. Female partner 16: “The trajectory will take a while and even then there is no guarantee that you get pregnant. What should we do if that happens?”

An important disadvantage regarding PGT was the expected physical and psychological strain of an IVF treatment including success related uncertainties and the influence of hormones on the women’s health. Women with NF1 (*N*=2) and cystic fibrosis (*N*=1) worried about the influence of the IVF treatment on their own health. Female partner 14: “I am worried about my health; I do not know how the hormones will influence my disease.” One couple mentioned the necessity to undergo an IVF treatment while being normally fertile and the loss of the sense of romance and control towards a pregnancy as disadvantages of the PGT trajectory. Female partner I4: “It is a heavy trajectory while you are normally fertile and the whole romantic idea of getting pregnant is gone. Suddenly a ‘white coat’ is involved.” Two couples with fertility problems related to the hereditary disease already knew that they needed an IVF treatment to establish a pregnancy, and therefore, PGT was perceived as a minor addition to this process.

Another disadvantage of PGT mentioned by several couples is that there is still a risk of having a child with the hereditary disease despite the complex procedure. Female partner 5: “They cannot guarantee you that they can exclude the disease, maybe for 99.99 percent but still, there is a chance.”

### PND

The main reason to opt for PND was that couples could achieve a natural pregnancy and can protect the child from being born with a genetic disease as they can opt to terminate a pregnancy. Couples indicated that PND enables them to prevent that their child will suffer. Male partner 14: “The choice is very difficult, but in the end you choose to give birth to a healthy child.”

Especially compared to PGT, the time to establish a pregnancy was considered as important. Couples also indicated that they prefer to try to conceive naturally. Male partner 6: “You [female partner] became pregnant quickly the first time and the next time. Time is a factor. We are not in a hurry, but it is nice if the trajectory would not last for years.” They also mentioned a psychological reason that PND avoids feelings of guilt towards their child for burdening them with the disease as they can terminate the pregnancy if the child is affected. Female partner 10: “That we never have to think ‘what if’ or regret it.”

The uncertainty when waiting for the prenatal test results was perceived as a major psychological disadvantage of PND. Female partner 10: “Are you happy with your pregnancy or aren’t you? You still don’t know.” Additionally, a main concern regarding PND was the question whether or not to terminate the pregnancy when the test results would show that the child has the disease. The deliberation regarding whether or not to terminate a pregnancy in case the fetus is affected was perceived as physically and emotionally difficult. Female partner 6: “It is nerve-racking. The risk of the miscarriage, waiting for the results and thinking about termination of pregnancy, which we already had once, which is very heavy.” Termination of pregnancy was acceptable for couples who perceived the hereditary disease as severe and/or if the disease would lead to mortality.

Lastly, the chance of a miscarriage due to invasive testing was an important reason to refrain from PND for several couples. Female partner 15: “I would doubt to do it because of the miscarriage. Even though it is only a small risk, but since I experienced it, I don’t want this to happen again.”

### Natural conception without genetic testing

The most frequently mentioned motive by couples to opt for natural conception without genetic testing was that in this way couples could avoid a medical trajectory. Relatedly, they perceived this option as the fastest way to establish a pregnancy compared to other reproductive options. Female partner 8: “Otherwise you have to wait for a year, and if the treatments are without result, you are just unlucky.” Compared to PND, a perceived advantage was avoidance of the extra risk of a miscarriage with PND and avoiding the stress and uncertainty associated with PND.

The severity and the transmission risk were also taken into account in the consideration to opt for natural conception without genetic testing. Female partner 16: “We have a 50% chance that the child will be healthy. And even if the child will be affected, it can have a good life.” However, chances were perceived differently. Male partner 6: “Healthy or not, it doesn’t matter how big that chance is. If it is 3% or 90%, it is nerve wrecking every time. Chances are difficult to understand.”

Some couples also felt confident in raising a child with the condition through their own experiences with the disease. Female partner 15: “In the meantime, you also learn how to deal with your child’s condition.”

Couples were especially reluctant to opt for a natural pregnancy when the hereditary disease was fatal or if it was a disease with variable expression in individuals (i.e., different clinical features among people with the same genotype) as they feared for a severe expression in their child. Female partner 3: “Maybe they should have a surgery, that is manageable. However, it is also possible that they will be born with bad skeletal abnormalities. You don’t know. There are so many different expressions regarding this syndrome.” Couples who experienced a neonatal death stated that they did not ever want to experience this again and therefore they would not opt again for a natural conception without genetic testing.

Couples also mentioned the psychological strain emerging from the uncertainty whether the child is affected or not as a disadvantage of natural conception. Male partner 1: “You would have nine months of stress, would the child be sick? If I have not gone completely mad during pregnancy, then I will go mad within two weeks after birth because of uncertainty.” Additionally, feelings of guilt towards the child were of major importance for some couples in the decision to refrain from natural conception without genetic testing. Male partner 16: “The feeling that there is a possibility to prevent it, but you don’t grab it. Guilt.”

### Donor gametes

Couples’ reasons for opting for donor gametes varied. Consideration of this option was mostly based on the notion that it can prevent the gene being transmitted to their offspring and that not all couples were eligible for PND or PGT. Furthermore, the use of donor gametes was generally perceived as one of the last possibilities to fulfill their child wish. Male partner 9: “At one moment, the question is: do you want a child or not? It doesn’t matter anymore if it is genetically related to both of us.”

Perceived advantages, especially compared to adoption, were that at least one of the parents is genetically related to the child, that the mother will carry the pregnancy, and that they will have the child from birth. Male partner 1: “A child of mine grows in her belly, it feels natural. It is not something strange, it is ours.”

However, the majority of couples considered the fact that only one of the parents is genetically related to the child as a disadvantage and therefore they did not consider the use of donor gametes as an option. Most couples would only consider the use of donor gametes if medical procedures would not lead to a successful pregnancy. A major practical disadvantage was the difficulty of finding donor gametes in the Netherlands. Couples indicated that they could ask friends, family, or visit a clinic. However, asking friends or family was perceived as very difficult as they had concerns regarding the consequences for their relationships. Male partner 1: “Within the family it is hard to find one. You could ask friends, but that is a bit strange. What kind of situations would you get? Therefore, we registered on a waiting list for an egg bank in the Netherlands.”

As it is difficult to find donor gametes in the Netherlands, couples may visit commercial clinics in other countries. However, these treatments are very expensive which made couples reluctant to visit these clinics. Female partner 2: “It’s strange that you seem to pay for a child. That has more to do with the feeling that if you become pregnant in a natural way, there is no money involved.”

The unfamiliarity of the donor was an important source of anxiety and tension when considering the use of donor gametes. Couples indicated that the unpredictability of the child’s appearance caused them to not further pursue this option. They indicated that this may also profoundly affects their daily life as couples were afraid that they have to explain and justify their choice to others. They were not concerned about the medical condition of the donor.

### Adoption/foster parenting

Couples who considered adoption or foster parenting as a possible option mentioned that they had a strong child wish and that it was not important to them if a child would be genetically related to one or both partners. They emphasized the perceived importance of being able to give a child in need a “home.” Female partner 11: “There are children in this world who need a safe home and we can offer that to them.”

Two couples indicated that they considered foster parenting and adoption because they experienced severe complications during previous pregnancies and wanted to prevent further complications and health care and societal costs of medical treatments.

The majority of the couples mentioned that adoption was not an option or that it would only be considerable if all the other reproductive options would not lead to a successful pregnancy and a healthy child.

Uncertainty regarding the physical and mental health of the child was mentioned as one of the main reasons to refrain from adoption. Additionally, couples were worried about difficulties in the attachment between child and adoptive/foster parents. Female partner 3: “You are not sure about the mental and physical health of the child, you don’t know the medical background.”

Another concern was the uncertainty regarding the adoption process and its long duration. Female partner 7: “There is no guarantee that you can adopt a child if you start with the procedure, you don’t know if it will happen.” The main practical reason to refrain from adoption was the costs related to this procedure.

A disadvantage of foster parenting was that you never know when the child will go back to its parents and that this can happen anytime. Couples were hesitant as they were afraid that they would experience this as very difficult and this could intervene and disrupt the normal family situation.

### Refraining from having further children

Most couples indicated that refraining from having further children was no option for them at this moment. This was perceived as the last option to be considered if all the other options would be unsuccessful. Two couples abandoned the idea of having a child as other procedures were unsuccessful or undesirable.

The main reason to refrain from having children was that the couples were satisfied with their lives and that a possible pregnancy carried medical health risks. Female partner 13: “We have each other. That is the most important. We are very happy with each other and nothing will change about that if we won’t have children.” Another reason mentioned is that it would be better for the physical health of the woman, as pregnancy carried additional medical risks related to the genetic condition. These couples did not want to undergo any medical procedures and did not want to accept the risk in a natural pregnancy and therefore, refraining from having children was the only option. One couple indicated that they felt uncertain about their future and that there would be no children to take care of them in older age.

## Joint decision-making

All couples indicated that they had had multiple conversations with each other before deciding on a reproductive option. These conversations were often unplanned and spontaneous. Some couples indicated that they had different preferences and that they were reluctant to consider the preferred reproductive option of their partner. Sometimes, multiple conversations and time to reconsider the options were needed for them to come to a decision that fitted the needs and preferences of both partners.

Most men and women indicated that their decision was mainly rational, meaning that it was based on facts and knowledge regarding the different reproductive options (*N*=13). Ten participants indicated that their decision was mainly emotional, indicating that they decided on an option primarily based on what felt right to them. Eight participants indicated that their decision was rational as well as emotional. There were no differences between men and women regarding the extent to which decisions were based on rational thoughts, emotions, or both. There were differences regarding the difficulty of the process; overall, women perceived the process more often as difficult compared to men.

When asked about the roles each partner took in the decision-making process, it seemed that women had a higher informational need. In addition to the information provided during consultations, most women searched extra information on the reproductive option online. Several men, however, indicated to take on a more active role during consultations with the clinical geneticist, meaning that they perceived that they asked more questions and took the lead.

Although in all couples, both partners supported the reproductive decision they made, significant differences in role taking were reported. Two main themes emerged, i.e., (1) the influence of gender, the fact that women should carry a pregnancy and undergo treatment, and (2) feelings of guilt associated with being a carrier.

Regarding the first theme, the influence of gender, 9 out of 16 couples agreed that the woman’s influence in the decision was stronger. Some male partners explicitly placed lower relative weights on their own versus their partner’s right to decide. The main reason mentioned was that the woman bears the physical burden of a pregnancy and therefore deserves to have a larger say in their ultimate decision. Male partner 4: “I immediately said: it is her body, it will be our child but it is her body. She needs to use hormones and needs to undergo surgery. I think she can decide for 70% and I can decide for 30%.”

Additionally, male partners mentioned that the woman bears the physical burden of the possible medical treatments. Male partner 1: “I believe that egg donation revolves around the woman. As a man, you only have a small contribution. So yes, I didn’t think I could make the decision.”

In 7 out of 16 couples, both partners indicated that they had decided together and both partners took equal part in decision-making. In none of the couples, the male partner had the final say.

Secondly, feelings of guilt were often mentioned. In 5 couples, both partners were carrier of a mutation. In couples where only one partner was carrier, several of the carriers mentioned feelings of guilt towards their partners. Male partner 16: “If we would start with the PGT trajectory, she needs to undergo it because of me. And what do I need to do? Nothing.” Especially male partners felt guilty that their genetic disease complicated the realization of their child wish and that their partner had to undergo a medical procedure and that their disease disrupted a natural pregnancy process. Male partner 10: “It is in my blood, I have the disease. I am the culprit.”

## Discussion

This study presents an overview of motives and considerations of couples at risk of transmitting a genetic disease to their offspring. Couples often experienced this reproductive decision-making process as difficult. All couples had multiple unplanned and spontaneous conversations to discuss their preferences and decide on which reproductive options to choose. Most couples, including those that opted for adoption, foster parenting, or the use of donor gametes, expressed a preference for the options in which a child is genetically related to both partners as is the case in natural pregnancy without genetic testing, PND and PGT. Other options such as the use of donor gametes, adoption, and foster parenting only qualified for consideration for a few couples. Refraining from having children was generally only considered if the other options would not lead to a successful pregnancy, if it could have a negative effect on the woman’s health or if the couple had ruled out foster parenting and adoption as an option.

Different motives and considerations and different views on the reproductive options were explored. The main motive to opt for PGT was the desire to have a healthy child which is in line with results of previous studies (Genoff Garzon et al. 2018). A previous study on couples with a balanced chromosomal rearrangement showed that this group perceived PGT as the fastest way to establish an ongoing pregnancy (De Krom et al. 2015; De Krom et al. 2020). However, in our study, couples mainly opted for a natural conception without genetic testing to avoid a medical trajectory and they perceived this as the fastest way to establish a pregnancy. Several couples considered PND as an option. The main motive to opt for PND was to achieve a natural pregnancy and to protect the child from the mutation if they opt for a pregnancy termination. Previous studies indicate that acceptability of PND varies strongly, with a majority of breast cancer gene (BRCA1/2) mutation carriers finding it a too drastic measure to prevent HBOC, but couples with Huntington Disease overall finding PND acceptable, even if they had to terminate the pregnancy (Derks-Smeets et al. 2014; Dommering et al. 2017). A tentative interpretation could be that the difference between the groups can be explained by the perceived severity of different hereditary diseases and the risk of transmitting a genetic disease (Garvelink et al. 2019). As costs of PND and PGT are reimbursed by the health insurance system in the Netherlands, we do not expect that costs play a role in their choice which is different to countries where costs are not covered (Genoff Garzon et al. 2018).

Donor gametes were often perceived as the last opportunity to fulfil their child wish without transmission of the gene to their offspring. Couples were concerned about the unfamiliarity of the donor and the child’s appearance. Couples who considered adoption or foster parenting had a strong child wish and had no preference for a genetically related child. However, in general, many couples expressed preference for genetically related offspring (Quinn et al. 2010). Most couples indicated that refraining from having children was considered as the last option and only if all the other reproductive options would not lead to a successful outcome. The main reason to refrain from having children was that the couples were satisfied with their lives and that a possible pregnancy carried medical health risks. It seemed that they accepted a life without children.

Couples emphasized the importance of supporting the same reproductive option. Couples indicated that their partner is the most important person in their decision-making process and that no other people close to them had significantly influenced their decision. The influence of gender and being a carrier were main themes in the decision-making process. Being a carrier posed feelings of guilt towards their partner, especially if a male partner was the carrier. This in line with a previous study in which male partners indicated that they felt guilty that their partner should undergo medical treatments (Derks-Smeets et al. 2014). Women appeared to have a greater impact in the decision-making process than men, which seemed to be related to the fact that the women will bear the pregnancy and she should undergo potential medical treatments. In a previous study on cystic fibrosis carriers, male partners also acknowledged that a woman should have the final say in reproductive decision-making. Reasons given were the fact that women act as a primary care provider and the woman should bear the pregnancy and physically go through related processes including testing and pregnancy termination (Laberge et al. 2019). This study only focused on the experiences of opposite-sex couples. The decision-making process of same-sex couples may be different as they possibly face other challenges during their decision-making process and the fulfillment of their child wish.

More research on joint informed decision-making is necessary to provide further insight into the joint decision-making process of decisional partners and their needs and wishes with regard to decision support. Most studies on prenatal or reproductive options only focus on the role of (expectant) mothers (Gee et al. 2017). If both partners participate in a joint decision, it is more likely that they explore more options as they can share their perspectives. This may lead to better reproductive health outcomes (Osamor and Grady 2018). Therefore, we have added a communication exercise to our DA which stimulates partners to deliberate together and talk about their values and the reproductive options. In this exercise, partners can write down values they personally perceive as important and compare and discuss these with their partner.

This study showed that the majority of the couples were not aware of all reproductive options. It is important to not only provide general, factual information about all reproductive options and their risks to couples but also provide information about important motives and considerations (e.g., practical, ethical) as a reproductive decision is highly personal and challenging. Therefore, a tailored individual and paired interactive approach in decision aids can be very helpful to optimize the quality of the reproductive decision and the decision-making process. A decision aid can provide factual information but can also ensure that people feel are more aware about their values and that they have a more active role in decision-making (Stacey et al. 2017).

Previous research has also shown that participants often report that information about reproductive options was not well discussed during consultations by health care providers (Quinn et al. 2010). Another study on BRCA1/2 mutation carriers showed that some of these couples refrained from having children because they did not know that options exist to prevent transmission of the disease (Quinn et al. 2010). The motives and considerations of couples are very personal and differ per couple even among couples at risk of the same hereditary disease or diseases that have the same transmission pattern. Therefore, it is important to not only provide general, factual information about all reproductive options and their risks to couples but also discuss couples’ individual motives and considerations relevant for specific diseases (e.g., concerns regarding the potential influence of ovarian stimulation on cancer risks) as a reproductive decision is highly personal. This highlights the importance of tailored decision support for couples at risk of transmitting a genetic disease to their offspring.

Several methodological aspects are worth mentioning. First, the couples were interviewed simultaneously. This may have influenced the discourse of the interview and the responses of the partners. This could have affected the results, particularly the findings with regard to joint decision-making. However, couples were encouraged to express their own opinion and their own view on different aspects of the reproductive decision-making process. We opted to conduct simultaneous interviews, as this research topic is very sensitive and personal. Couples often share painful experiences; simultaneous interviewing allowed them to contextualize these experiences together and comfort each other (Zarhin 2018). Individual interviewing as well as joint interviewing of couples has disadvantages and advantages; however, reproductive decision-making is a shared experience and therefore joint interviewing can provide specific insights in the decision-making process of the couples. This study included a small group of participants and this affects the generalizations that can be made. However, this is one of the first studies to provide insight into a heterogeneous group of couples that have considered and opted for different reproductive options.

This study provides insight in the most important motives and considerations of couples at risk of transmitting a genetic disease to their offspring and a child wish. Additionally, it provides a first insight into the joint decision-making process of these couples. The findings have merit for further research to support counseling and decision support for both partners. This is important as couples indicate that a need for support during the reproductive decision-making process as this complex decision can have major consequences in their lives (Gietel-Habets et al. 2018).

## Data Availability

The datasets generated during and/or analyzed during the current study are not publicly available.
